# Clinical and histopathological investigation of the possible occurrence of tracheobronchial disease in cats with chronic gingivostomatitis

**DOI:** 10.3389/fvets.2025.1624016

**Published:** 2025-06-26

**Authors:** Olga Lorida, Alexandros Konstantinidis, Georgia D. Brellou, Georgia Koutouzidou, Paraskevi Papadopoulou, Apostolos Matiakis, Katerina K. Adamama-Moraitou, Serafeim Papadimitriou

**Affiliations:** ^1^Companion Animal Clinic, Faculty of Health Sciences, School of Veterinary Medicine, Aristotle University of Thessaloniki, Thessaloniki, Greece; ^2^Laboratory of Pathology, Faculty of Health Sciences, School of Veterinary Medicine, Aristotle University of Thessaloniki, Thessaloniki, Greece; ^3^Department of Agriculture, School of Agricultural Sciences, University of Western Macedonia, Florina, Greece; ^4^Laboratory of Diagnostic Imaging, Faculty of Health Sciences, School of Veterinary Medicine, Aristotle University of Thessaloniki, Thessaloniki, Greece; ^5^Department of Oral Medicine and Pathology, School of Dentistry, Aristotle University of Thessaloniki, Thessaloniki, Greece

**Keywords:** oral inflammatory disease, feline chronic gingivostomatitis, tracheobronchial disease, bronchial secretions, lower respiratory tract

## Abstract

**Introduction:**

Feline chronic gingivostomatitis (FCGS) is a debilitating and highly painful inflammatory disorder of the feline oral cavity. Evidence suggests that feline chronic gingivostomatitis (FCGS) induces systemic effects that extend beyond localized oral pathology, contributing to overall health decline in affected cats. The aim of this study was to investigate the potential impact of FCGS on the lower respiratory tract.

**Methods:**

This is a prospective study, that included 42 cats with clinical signs of FCGS and five healthy control cats exhibiting no signs of oral disease. All cats underwent physical, oral, and endoscopic examinations of the lower respiratory tract. Radiological evaluation of the thorax was also performed. Lesions in the respiratory tract detected upon endoscopy and the oral cavity were recorded and scored. In cats with FCGS biopsies from bronchial mucosa were obtained from sites showing endoscopic evidence of inflammation.

**Results:**

Respiratory lesions were identified in all FCGS cats included in the study. Specifically, secretions were detected in 42 out of 42 (100%) cats, bronchial mucosal edema in 33 out of 42 (78.6%), a granular appearance in 14 out of 42 (33.3%), and hyperemia in 11 out of 42 (26.2%). Histopathological examination revealed mucosal and submucosal inflammation in 30 out of 36 (83.3%) cats and mucosal edema in 25 out of 36 (69.4%). Additionally, fibrosis was observed in 25 out of 36 (69.4%) samples, hyperplasia, or dilatation of bronchial glands in eight out of 36 (22.2%), and vascular wall thickening in 11 out of 36 (30.5%). Bronchial smooth muscle hypertrophy was present in 22 out of 36 (61.1%) examined samples. An attempt to correlate oral and respiratory lesion severity found no statistically significant correlation between stomatitis index, tracheobronchoscopy, or histopathological scores.

**Discussion:**

FCGS appears to coexist with lower respiratory tract disease. During FCGS management, it might be essential to address any underlying respiratory disorder, as it may favor the outcome of the primary disease, while remaining unattended it may increase the likelihood of FCGS recurrence.

## Introduction

Feline chronic gingivostomatitis (FCGS) is a highly painful and severe inflammatory condition that affects the oral cavity of cats. It presents as either diffuse or localized lesions, which may be ulcerative or hyperplastic, with variable distribution throughout the oral cavity (1–6). Clinically, cats with FCGS exhibit reduced appetite, excessive drooling, halitosis, and significant pain, often leading to behavioral changes, including decreased grooming and social interaction (3, 4, 7, 8). Histopathological studies have shown that FCGS lesions are characterized by a pronounced lymphoplasmacytic infiltration, while neutrophils and mast cells are less frequently observed (3, 5, 7). Transcriptomic analyses indicate that the inflammatory response is significantly mediated by interleukin-6 (IL-6), suggesting a crucial role for this cytokine in the disease's pathogenesis (6, 8–10). Although the exact cause of FCGS is unknown, it is believed to involve an abnormal immune response (1, 2, 5, 11), possibly triggered by oral microbial antigens (3, 12, 13).

Due to the anatomical continuity between the oropharynx and the lower respiratory tract, the associated oropharyngeal microbiome or its metabolites may extend to other parts of the respiratory system, including the lungs (14). To maintain respiratory sterility, several physical and immune defense mechanisms—such as the mucociliary apparatus, alveolar macrophages, and a tightly regulated immune system—work concurrently (14–16). In human medicine, inflammatory diseases of the oral cavity, such as periodontitis, have been reported as important causes of systemic disorders, including respiratory diseases (17). This occurs due to the aspiration of oral secretions into the lungs, making periodontitis an important source of lung infections (17–19). Additionally, salivary enzymes from infected periodontal tissues contribute to the pathogenesis of respiratory inflammation (18).

Approximately 30% of cats with FCGS are resistant to recommended treatment (1, 8, 20). It is assumed that the coexistence of respiratory inflammation and the influence of pro-inflammatory cytokines on the oral mucosa and respiratory system may trigger immunological mechanisms that exacerbate FCGS and affect its outcome. The primary aim of the present study was to investigate the potential impact of FCGS on the lower respiratory tract and to describe the lesions detected during endoscopy and histopathology, an area not previously explored in the international literature. Additionally, an attempt was made to assess the correlation between the severity of both conditions.

## Materials and methods

### Study population

This study was approved by the Ethics committee, School of Veterinary Medicine (Approval No: 199866(810)/14/4/2021). All participating cat owners signed a written form allowing their pets to undergo general anesthesia and participate in the study. The study included 42 client-owned cats with FCGS, along with five healthy cats admitted for ovariohysterectomy or orchiectomy. A detailed history was meticulously recorded for each cat, with particular attention to clinical signs, including the manifestation patterns of oral pain, anorexia, and salivation. Additionally, any information concerning prescribed medication, such as antibiotics, steroids, non-steroidal anti-inflammatory drugs, and treatment for tracheobronchial disease was also recorded. Both groups underwent a comprehensive physical and oral examination. To further assess their health status, a complete blood count (CBC) and serum biochemistry were performed.

### Inclusion and exclusion criteria

Cats with FCGS displaying oral symptoms both at the time of admission and prior to tracheobronchoscopy and surgery were requires to be off medication for at least 15 days (45 days for those previously treated with methylprednisolone acetate). FCGS was confirmed through both macroscopic and histopathological examination. These cats had no history of upper or lower respiratory disease and showed no clinical signs suggestive of respiratory involvement at the time of evaluation. The control group included young, healthy, and adult cats without any visible oral pathology or prior medical history of upper or lower respiratory disease. Cats with concurrent diseases, such as cardiac disease, immune-mediated disorders, neoplasia, upper and lower respiratory tract infections, urinary tract infection, and diabetes mellitus, were excluded from the study. These conditions were ruled out through thorough investigation of previous medical records, current owner history, physical examination findings, as well as complete blood count (CBC) and serum biochemistry examination. Feline leukemia virus (FeLV) and feline immunodeficiency virus (FIV) status were evaluated using a Snap FeLV/FIV Combo Test (IDEXX).

### Study design

Tracheobronchoscopy was consistently conducted prior to major surgery in both groups. Food was withheld for 12 h and water for 2 h before general anesthesia. The animals were anesthetized by the same anesthesiologist and the anesthetic protocol was identical for all cats: acepromazine maleate (0.02 mg/kg, Acepromazine, Alfasan, Nederland B.V.) and butorphanol (0.5 mg/kg, Dolorex, Intervet International, Holland) intramuscular for premedication, followed by propofol (Propofol MCT/LCT/Fresenius 1%, Fresenius Kabi, Greece) intravenous for total intravenous anesthesia until the tracheobronchoscopy was completed. Before thracheobronchoscopy, thoracic radiography (ventrodorsal, right lateral, and left lateral views) was performed and evaluated by a single board-certified radiologist who was blinded to the medical history of the cats. Oxygen was supplemented to each cat with an oxygen mask before tracheobronchoscopy and was continued throughout the procedure through the endoscopy channel. During tracheobronchoscopy, total intravenous propofol to effect was administered to maintain an appropriate anesthetic depth. After the tracheobronchoscopy, cats were intubated and connected to an isoflurane system with 100% oxygen supply.

### Tracheobronchoscopy

Tracheobronchoscopy was conducted using a sterilized Olympus BF-PE2 flexible bronchoscope (Olympus Corp., Tokyo, Japan). The bronchoscopic appearance of the bronchial mucosa lesions like hyperemia, edema, and mucosal granularity, along with their distribution and the presence and type of secretions were recorded. A modified scoring system based on Thompson et al. (21) and Kavarnos et al. (22) was applied to assess hyperemia, edema, and granularity of the bronchial mucosa, as well as the amount of bronchial secretions. Scoring ranged from 0 to 3, based on the presence and severity of lesions for each cat, while mucosal granularity was scored as either present (1) or absent (0). Lesion distribution was categorized as localized (1) or diffuse (2). The overall bronchoscopic appearance of the bronchial mucosa for each cat was calculated by summing these points, resulting in a total endoscopic score ranging from 0 (normal) to 18. According to the severity of endoscopic lesions, the cats were classified into four groups: normal (0), mild (1–6), moderate (7–12), and severe (13–18).

Following macroscopic inspection, two biopsy samples of the bronchial mucosa were collected from cats with FCGS. The samples were fixed in 10% neutral buffered formalin and then sent for histopathological examination. Paraffin-embedded tissue blocks were sectioned at 5 μm thickness and stained with hematoxylin and eosin. A single pathologist performed a blinded histopathological examination. A structured scoring system was developed to better reflect the observed pathological findings. According to this scoring system, the bronchial mucosa was evaluated for inflammatory cell infiltration, fibrosis, and edema, as well as for bronchial gland hyperplasia and thickening of the vessel walls and bronchial smooth muscle. Each parameter was scored as absent (0) or present (1). Inflammatory cell infiltration, fibrosis, and bronchial mucosal edema were further graded based on their extent and severity as follows: 1 (mild) and 2 (moderate). The total histopathological score for each cat was calculated by summing these individual parameters, resulting in a cumulative score ranging from 0 (normal) to 12. Based on the overall severity of histopathological lesions, cats were classified into three groups: normal (0), mild (1–6), and moderate (7–12).

### Oral examination

Cats from both groups were assessed using the periodontal disease stage (23). Furthermore, all FCGS cats received a detailed dental examination, including the modified Stomatitis Disease Assessment Index (SDAI) (24). The SDAI measures the severity of oral inflammation as evaluated by the veterinarian, captures the owners' perceptions of the syndrome's impact on their cats, and combines these two indicators into a score sheet. Subsequently, intraoral radiographs were taken for all cats with FCGS (Progemy, Midmark Corporation, U.S.A). Before the surgical procedures, such as partial or full mouth extractions and CO_2_ laser treatments, biopsies were taken from the palatoglossal folds area. These specimens were prepared and fixed similarly to the respiratory specimens and examined blindly by the same pathologist. Based on the criteria by Harley et al. (25), histological samples from the oral mucosa were graded as follows: Grade 0: normal, Grade 1: mild inflammation, Grade 2: moderate inflammation, and Grade 3: severe inflammation. Post-surgery, the medications administered were specific to each cat's requirements and included meloxicam (0.1 mg/kg, Metacam, Boehringer Ingelheim Animal Health, Canada), tramadol (2 mg/kg, Tramal, Grünenthal GmbH, Germany), buprenorphine (0.02 mg/kg, Bupredine, Dechra Pharmaceuticals, United Kingdom), and clindamycin (10 mg/kg, Clindamycin/Anfarm, Anfarm Hellas S.A., Greece). Based on the tracheobronchoscopical appearance of the respiratory tract observed during tracheobronchoscopy, doxycycline (10 mg/kg, Ronaxan, MERIAL, France) was administered if needed (26).

### Statistical analysis

Both descriptive and inferential statistics were applied. Linear regression coefficient (*b*) and the Pearson correlation coefficient (*r*) were estimated to evaluate the linear relationship between two continuous variables, i.e., FCGS and tracheobronchoscopy scoring, as well as histological bronchial and oral mucosa scoring. The linear regression coefficient quantifies the relationship between predictor variable and the response in a regression model. The Pearson coefficient quantifies the degree to which changes in one variable predict changes in another, based on their covariation. The coefficient indicates the strength and direction of the relationship and ranges from −1 (strong negative correlation) to 1 (strong positive correlation) (27). The statistical analysis was performed using SPSS (Version 29, provided by the Aristotle University of Thessaloniki, Greece) and significance was declared at ≤ 0.05 significance level. To specifically investigate the correlation between the clinical severity of oral inflammation and respiratory findings, the SDAI scores, as well as a modified bronchoscopic scoring system of the respiratory tract, were incorporated into the statistical analysis. Pearson correlation coefficients were calculated to evaluate associations between SDAI clinical scores and both bronchoscopic and histopathological scores of oral and respiratory mucosa. This integrative approach combined clinical and pathological data to thoroughly assess potential links between oral and respiratory disease in cats with FCGS.

## Results

### Demographic data

Among the 239 cats presented to the clinic with FCGS, between March 2021 and October 2022, 42 met the inclusion criteria in order to be included in the study population, of which 24/42 (57.1%) were male and 18/42 (42.9%) female. Purebred cats represented a minority, comprising 2/42 (4.8%) cases, including Maine Coon 1/42 (2.4%) and Siamese 1/42 (2.4%). The remaining 40/42 (95.2%) cats were European Shorthair Breed. The median age at presentation was 5 years (range 1–12 years), with a median body weight of 4 kg (range 2–6.6 kg). Among 42 FCGS cats included in the study, FeLV infection was detected in 18 cats, while FIV infection was detected in 10 cats.

Five healthy cats were included in the control group, of which 4/5 (80%) were male and 1/5 (20%) female. Control group cats were European Shorthairs. The median age at the presentation was 3 years (range 2–4years), and their median body weight was 4 kg (range 3.6–5.1). Among the five healthy control cats, two were FeLV-positive and one tested positive for FIV.

### Thoracic radiography findings

Thoracic radiographs of all FCGS cats in this study revealed mild bronchial pattern lesions, whereas no pathological signs were observed in the control group. In all examined cases, peribronchial infiltration was recognized in both right and left lateral recumbency. This was clearly visible in end-on views as a soft tissue cuff around the affected bronchus, giving the characteristic “doughnut” appearance. These areas of infiltration, along with occasional bronchial calcification, were most commonly observed in the upper lung fields. Except for the larger bronchi near the hilus, the bronchial pattern was predominantly evident in the upper caudal lung fields.

### Tracheobronchoscopy findings

**Figure 1:**
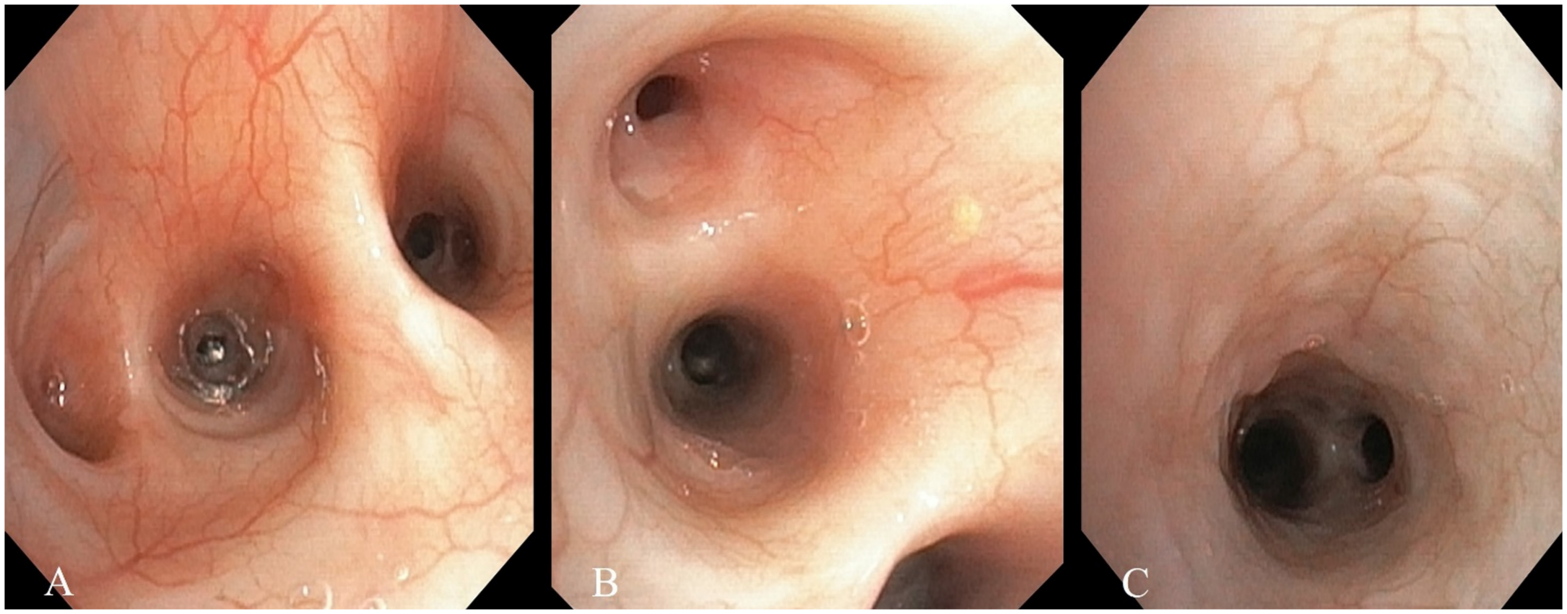
Bronchoscopy images of cats with feline chronic gingivostomatitis. **(A)** Generalized hyperemia of the bronchial mucosa and increased bronchial secretions. **(B)** Edema of the bronchial mucosa. **(C)** Granularity of the bronchial mucosa.

Tracheobronchoscopy was performed on all 42 cats diagnosed with FCGS and on five healthy control cats. Examination of the trachea revealed no macroscopic abnormalities in either group. In both healthy control cats and those affected by FCGS, the trachea appeared normal, apart from the consistent presence of secretions in the FCGS group. No macroscopic abnormalities of the lower respiratory tract were observed in the control group. In contrast, all cats with FCGS (100%) exhibited macroscopic lesions in the lower airways (Table 1), most notably characterized by the presence of increased bronchial secretions (Figure 1). The secretions were classified as serous, mucoid, or mucopurulent in nature. Examination of the trachea in FCGS-affected cats revealed no abnormalities apart from the presence of secretions, which were consistently observed in all cases. The severity of the bronchoscopic findings detailed in Table 2, with bronchial secretions and mucosal edema being the most common findings.

**Table 1:** Tracheobronchoscopic findings in 42 cats with feline chronic gingivostomatitis.

**Lesions observed by tracheobronchoscopy**	***n* (%)**
Bronchial secretions	42 (100)
Edema	33 (78.6)
Hyperemia	11 (26.2)
Granular appearance	14 (33.3)

**Table 2:** Classification of lesions observed via tracheobronchoscopy in 42 cats with feline chronic gingivostomatitis.

**Classification**	***n* (%)**
Mild	11 (26.1)
Moderate	28 (66.7)
Severe	3 (7.2)

The severity of macroscopic lesions of the respiratory system is presented in Table 2, with the majority of cats showing moderate lesions in 28/42 cats (66.7%).

No statistically significant correlation was observed between the intensity or chronicity of FCGS lesions and the severity of macroscopic abnormalities in the respiratory tract. The results of the correlation analysis (Table 3), which are consistent with those obtained from the regression analysis, revealed a negative correlation between the stomatitis index and tracheobronchoscopy findings (*r* = −0.176; *p* = 0.265). This *p*-value exceeds the conventional threshold for statistical significance (*p* < 0.05), indicating that the observed relationship is not statistically significant.

**Table 3:** Linear correlation results of the macroscopic findings in the oral cavity (stomatitis INDEX) and tracheobronchoscopy score, as well as between histopathological findings in the oral mucosa and bronchi.

**Variables**	**n**	**Pearson *r***	***p*-Value**
Stomatitis index—bronchoscopy score	42	−0.176	0.265
Bronchial score—oral mucosa	36	0.073	0.671

### Oral examination

**Figure 2:**
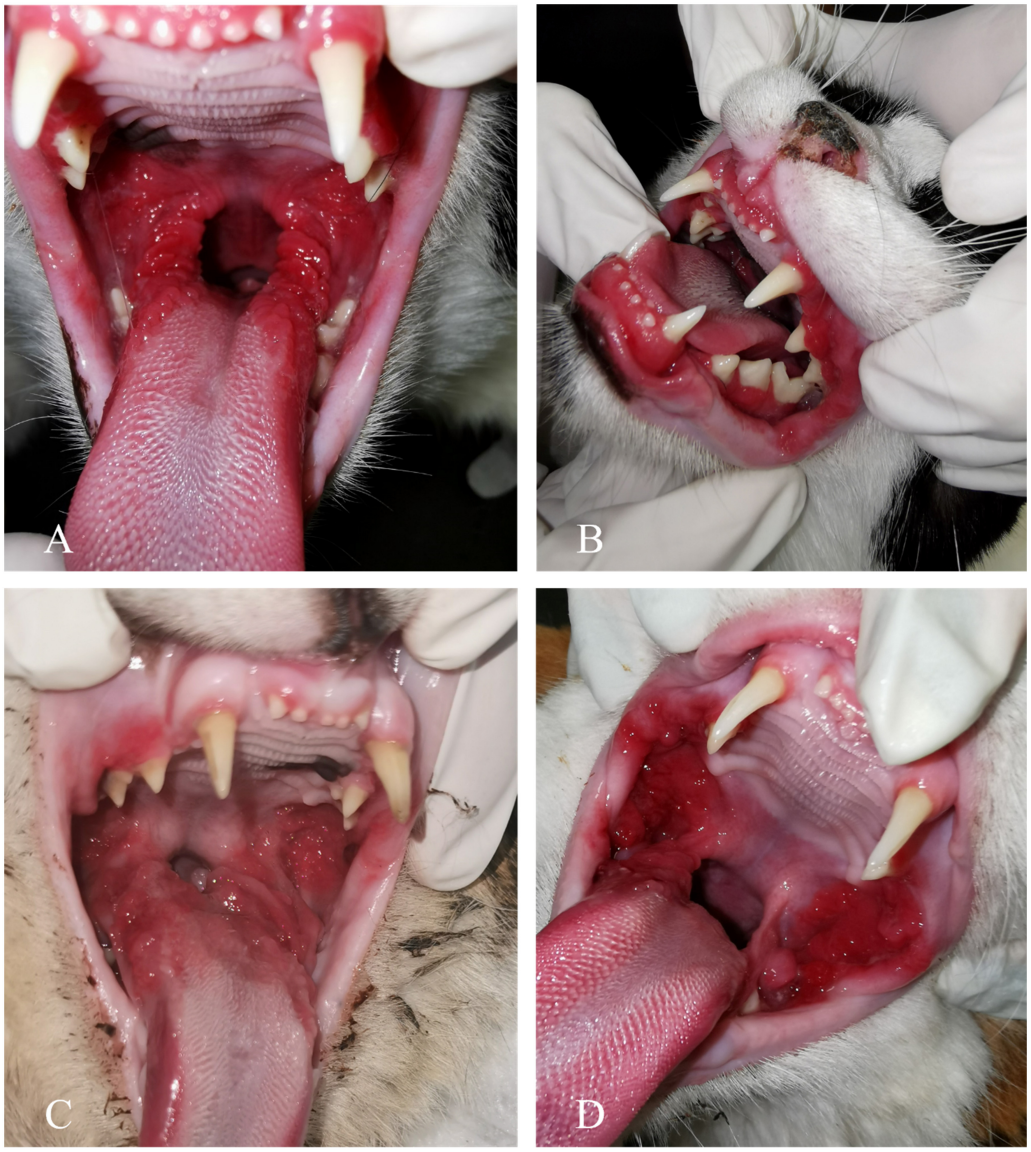
Clinical manifestation of feline chronic gingivostomatitis (FCGS). **(A)** A patient with refractory ulcerative FCGS. **(B)** Concurrent inflammation of the gingiva, alveolar, and buccal mucosa. **(C)** Ulceration and proliferation affecting both palatoglossal folds and sublingual mucosa. **(D)** A case exhibiting both ulceration and proliferation, with notable proliferation of the buccal mucosa.

An oral cavity examination was performed in all FCGS cats and control cats. The severity of lesions in FCGS cats was assessed using the SDAI scoring system, with severity grades determined as outlined in Table 4 (Figure 2) (24). In the cohort of 42 FCGS cats evaluated, moderate lesions were the most common, observed in 31/42 cats (73.81%), followed by severe lesions in 8/42 cats (19.05%) and mild lesions in 3/42 cats (7.14%).

**Table 4:** Classification of lesions observed in oral cavity in 42 cats with feline chronic gingivostomatitis.

**Classification**	***n* (%)**
Mild	3 (7.14)
Moderate	31 (73.81)
Severe	8 (19.05)

### Histopathological examination

**Figure 3:**
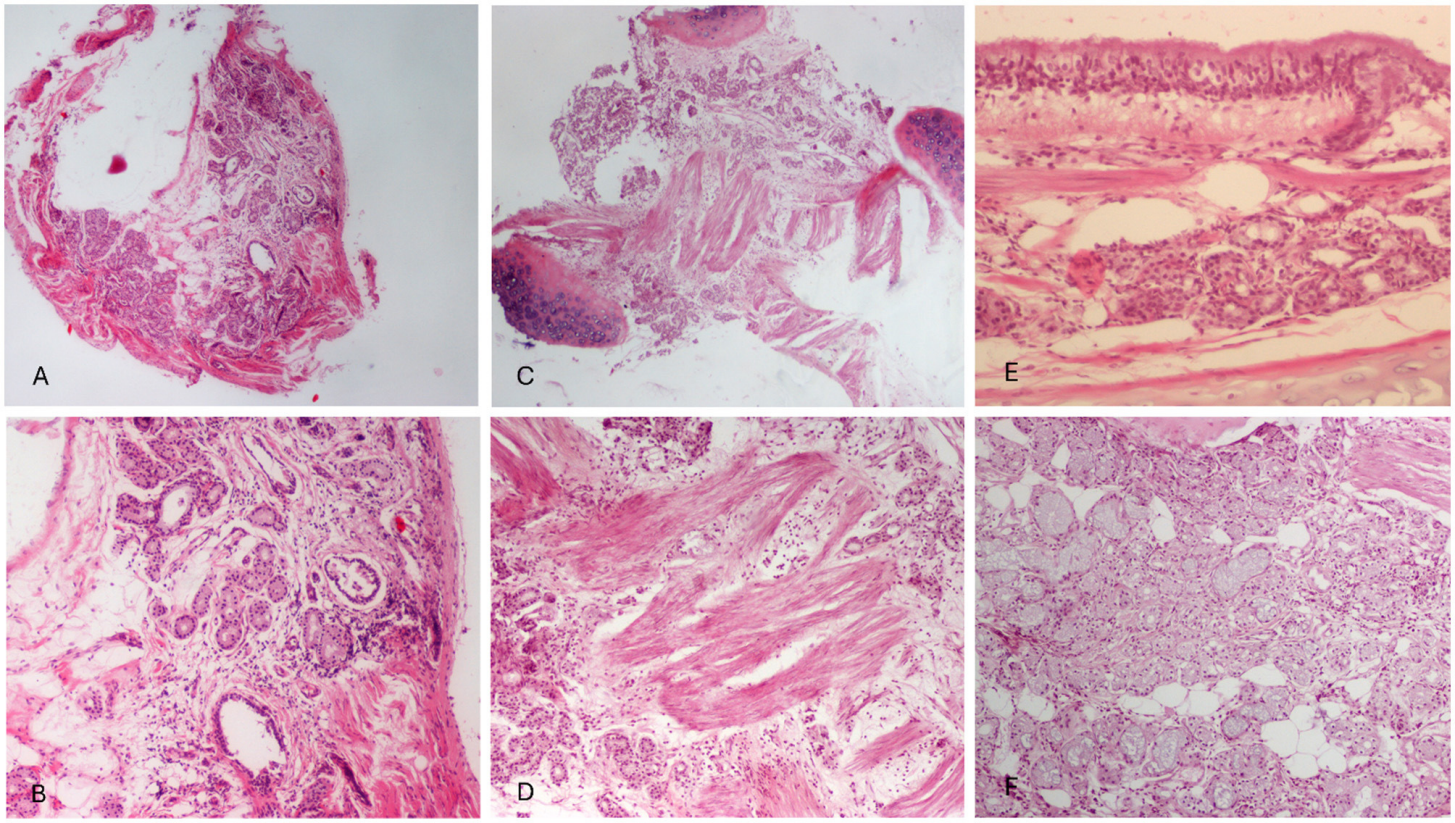
Histopathological findings of bronchial samples in cats with feline chronic gingivostomatitis. **(A, B)** Mild bronchial gland and smooth muscle hyperplasia, interstitial edema and inflammatory cell infiltration in the submucosa. Lymphocytes and plasmacytes predominate. Also a few glands show dilatation. **(C, D)** Moderate smooth muscle hyperplasia, with intersected bundles of muscle fibers and interstitial edema. Inflammatory cells, mostly lymphocytes as well as plasmacytes and neutrophils in the submucosa among bronchial gland acini and smooth muscle fibers. Dilated vessels are also observed. **(E)** Bronchial mucosa is characterized by intraepithelial and lamina proprial edema and mild mucosal and submucosal mononuclear cell infiltration. **(F)** Bronchial gland hyperplasia consisting mostly of foamy, mucous acinar cells with flattened nuclei. Foci with fibrosis, sometimes periacinar, and a few mononuclear cells are shown in the interstitial tissue. H-E, original magnification **(A, C)** 40×, **(B, D, F)** 100×, and **(E)** 200×.

Bronchial biopsies were obtained from all 42 FCGS affected cats, but 36 samples were successfully evaluated histopathologically due to technical difficulties in tissue handling (Table 5). Mucosal and submucosal inflammation was the most common finding, observed in 30/36 cats (83.3%). Mucosal and submucosal inflammatory cell infiltration, consisting predominantly of mononuclear cells (lymphocytes and plasmacytes) and/or neutrophils, was commonly observed. Intraepithelial lymphocytes were also identified in some instances. Mucosal edema and fibrosis were each present in 25/36 cats (69.4%). Mucosal edema was characterized by intracellular edema of the surface epithelium, occasionally accompanied by dissociation of epithelial cells, and by thickening of the submucosa due to interstitial edema, leading to separation of submucosal structures. Bronchial smooth muscle hypertrophy was detected in 22/36 cats (61.1%), vascular wall thickening in 11/36 cats (30.5%), and hyperplasia or dilatation of bronchial glands in 8/36 cats (22.2%). Based on histopathological scoring (Table 6), the majority of lesions were classified as mild (23/36, 63.9%), while moderate lesions were recorded in 13 cases (36.1%; Figure 3).

**Table 5:** Histopathological findings in the bronchial mucosa in 36 cats with feline chronic gingivostomatitis.

**Histopathological findings**	***n* (%)**
Mucosal and submucosal inflammation	30 (83.3)
Mucosal edema	25 (69.4)
Fibrosis	25 (69.4)
Hyperplasia or dilatation on bronchial glands	8 (22.2)
Vascular wall thickening	11 (30.5)
Bronchial smooth muscle hypertrophy	22 (61.1)

**Table 6:** Classification of histopathologic lesions of the bronchial mucosa in 36 cats with feline chronic gingivostomatitis.

**Classification**	***n* (%)**
Mild	23 (63.9)
Moderate	13 (36.1)

**Figure 4:**
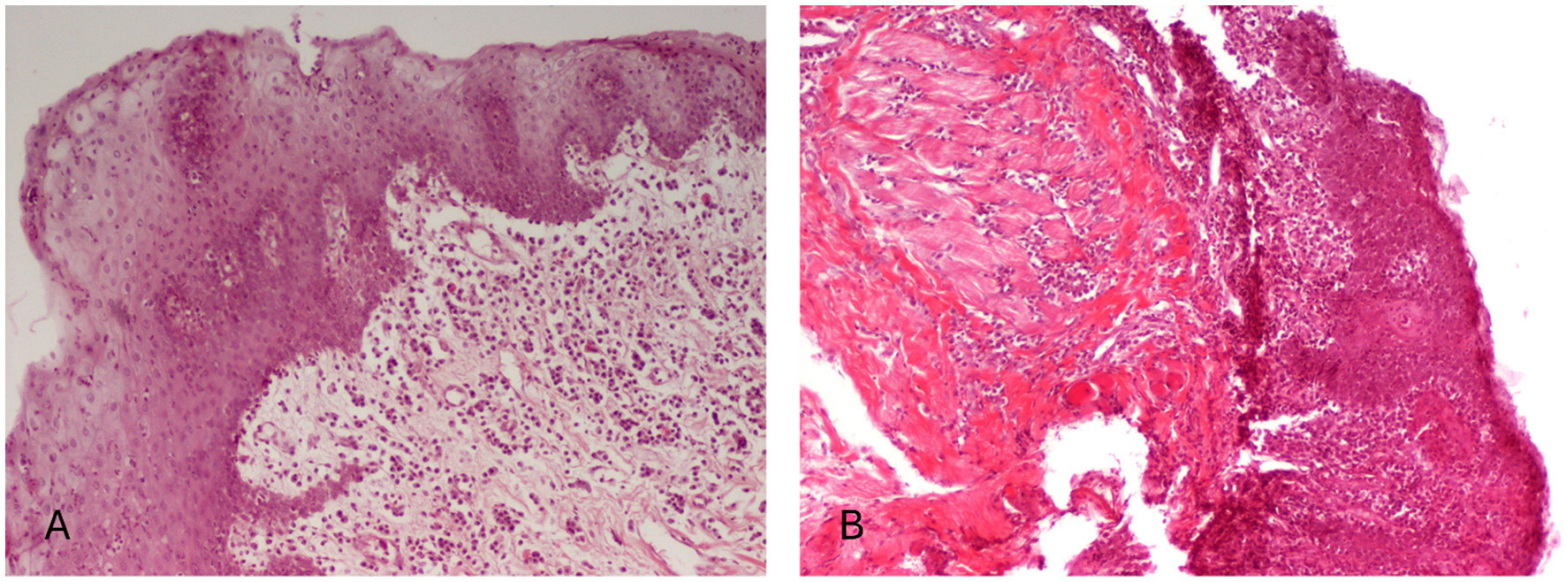
Histopathological findings in cats with feline chronic gingivostomatitis. **(A)** Hyperplasia of squamous epithelium with features of degeneration, a few intraepithelial lymphocytes and presence of sparse neutrophils among epithelial cells. In the lamila propria edema and inflammatory cell infiltration of moderate degree, consisting mostly of plasmacytes and lymphocytes are also seen. **(B)** The gingiva shows focal ulceration and stratified squamous epithelium hyperplasia and degeneration. Among epithelial cells, numerous inflammatory cells (mostly neutrophils, lymphocytes, and macrophages) are observed. Severe inflammatory cell infiltration, mostly lymphocytic/plasmacytic, expands to the lamina propria, the submucosa, as well as the adjacent degenerated striated muscle. H-E, original magnification **(A, B)** 100×.

Oral mucosal biopsies were collected from all 42 FCGS-affected cats. Histopathological lesion, based on the criteria described by Harley et al. (25), is detailed in Table 7. Severe lesions were the most commonly observed, affecting 23/42 cats (54.8%), while moderate lesions were noted in 14/42 cats (33.3%), and mild lesions in 5/42 cats (11.9%; Figure 4).

**Table 7:** Classification of histopathologic lesions of the oral mucosa in 42 cats with feline chronic gingivostomatitis.

**Classification**	***n* (%)**
Mild	5 (11.9)
Moderate	14 (33.3)
Severe	23 (54.8)

No statistically significant correlation was found between the histopathological severity of FCGS-associated oral inflammation and the degree of bronchial tissue alterations. These findings suggest that the extent of oral mucosal involvement in FCGS does not reliably reflect or predict the severity of concurrent lower airway changes. The correlation analysis results (Table 3), which are consistent with those from the regression analysis, showed no meaningful association between the tracheobronchial score and oral mucosal histopathology (Pearson's *r* = 0.073; *p* = 0.671). This near-zero correlation coefficient and non-significant *p*-value (*p* > 0.05) confirm the absence of a statistically significant relationship between these parameters.

## Discussion

The etiopathogenesis of FCGS remains controversial and is considered multifactorial (4, 12, 28). In this prospective study, respiratory lesions were investigated in 42 cats diagnosed with FCGS with the aid of tracheobronchoscopy and histopathological evaluation of bronchial mucosa. Five healthy cats were included as a control group. The primary tracheobronchoscopic finding in FCGS population was the increased bronchial secretions in 100% of cats. The most significant histopathological observation was mucosal and submucosal inflammation of bronchial mucosa in 83.3% of cats. Our findings suggest that the inflammatory component in the oral cavity of cats with FCGS, along with the observations of Kouki et al. (2), where esophageal involvement was reported in 98% of affected cats, a systemic effect maybe suspected.

The respiratory tract is safeguarded by a mucosal defense system composed of epithelial cells forming physical barriers. These epithelial cells possess cilia that move within a mucus layer, trapping particles and pathogens, facilitating their removal from the lungs (29). Additionally, the cough reflex aids in expelling inhaled foreign bodies (16, 30). The innate immune system contributes antimicrobial substances released by phagocytes and the airway epithelium, which, along with macrophages and neutrophils, work to neutralize microorganisms. Alveolar macrophages, essential cells in maintaining healthy airways, clear debris, and dead cells, thereby strengthening respiratory defenses (16, 31, 32). Adaptive immunity also plays a crucial role, with T-cells governing cellular immunity and B-cells producing antibodies to support humoral immunity. Together, innate and adaptive immune responses coordinate an integrated defense against lower respiratory system threats.

All cats with FCGS included in this study were evaluated for associated pathological findings in the respiratory system. Endoscopy of the respiratory tract revealed increased bronchial secretions in all cases, 42/42 cats (100%). In human medicine, bronchopneumonia due to aspiration of oropharyngeal secretions in Intensive Care Unit patients with mechanical aspiration events is relatively common, occurring in 23% of cases (15). In veterinary medicine, however, limited research exists on this topic; only one study reports a small percentage of dogs affected by bronchopneumonia due to aspiration following general anesthesia (33). Given this risk, appropriate pre-anesthetic treatment was selected to minimize bronchial secretions and ensure the safety of each cat during the procedure. Cats with FCGS often exhibit severe salivation, leading to the saliva accumulation in the oropharyngeal region (1). Excessive drooling may increase the risk of aspiration of oropharyngeal secretions in cats suffering from FCGS. However, according to owner report, none of the cats presented to the clinic had a history of lower respiratory disease. These findings highlight the potential relevance of respiratory system evaluation in cats with FCGS, even in the absence of overt clinical signs of lower respiratory disease.

In humans, another direct link between the respiratory system and oral inflammatory diseases, such as periodontal disease, is the migration of microbes from the oral cavity, which can trigger respiratory epithelial cells to secrete inflammatory markers and proteases (18, 19, 34, 35). Additionally, the microbiome present in saliva may contribute to the secretion of pro-inflammatory markers in respiratory epithelial cells in FCGS-affected cats following aspiration of these secretions (2, 13).

Literature suggests a link between the respiratory system and oral health through indirect mechanisms in humans. Notably, periodontitis, a condition that induces low-grade inflammation, appears to impact respiratory health (18, 19). Cytokines and chemokines generated in periodontal tissues can travel via the bloodstream and exacerbate respiratory diseases, primarily by contributing to endothelial dysfunction (19, 36, 37). According to Peralta et al. (8), the transcriptional profile of oral mucosal tissues in cats with FCGS reveals a heightened presence of genes and pathways involved in immune response and inflammation, with IL-6 playing a central role in these processes (5). Recently, a research has further examined IL-6 impact on chronic respiratory diseases and respiratory inflammation. Dawson et al. (38) indicate that IL-6 family cytokines are key immunity regulators, helping to eliminate pathogens and combat viral infections (38, 39). However, excessive activation of these cytokines can lead to pathological states such as fibrosis and hyperinflammation. IL-6 also promotes CD4+ Th2 and Th17 responses by modulating T-cell differentiation and is released by both immune cells and lung epithelial cells in response to allergens (40). In summary, elevated levels of these pro-inflammatory cytokines may influence the immune function in cats with FCGS as a whole, with the IL-6 family playing a critical role in respiratory immune responses.

Macroscopic findings from airway endoscopy indicated changes consistent with certain respiratory pathologies. According to a study by Johnson and Vernau (41), bronchial secretions were the most common finding in cats with lower respiratory diseases, observed in 83% of cases. Similarly, our research aligns with these findings, as 100% of cats with FCGS exhibited bronchial secretions. The second most frequent observation, found in 78.6% of cases, was mucosal edema. This edema appears to be a precursor to bronchial collapse, either preceding or following aspiration, and is also characteristic of inflammatory respiratory conditions such as asthma and chronic bronchitis in cats (41–43). In 33.3% of the cats included in the present study, a granularity of the bronchial mucosa was observed, aligning with key findings of Johnson and Vernau's research (41). Finally, hyperemia was also observed in 26.2% of cases. In contrast, tracheobronchoscopy in the control group cats revealed no significant lesions, but only a mild increase in bronchial secretions, primarily from the oropharynx at the end of the procedure.

Histopathological examination of the bronchial mucosa was successful in 36 out of 42 cats. Histopathological lesions were classified as mild in 23/36 (63.9%) and moderate in 13/36 (36.1%). Control samples were not obtained as there were no macroscopic lesions and ethical considerations precluded the sampling of healthy tissue. Mild chronic inflammation similar results in histopathological examination has been reported by Arafah et al. (44) and Cosio et al. (45) in humans suffering from chronic respiratory diseases. In the present study, inflammation of the mucosal and submucosal tissue of the bronchi was present, with a high incidence, observed in 30/36 cats (83.3%) aligning with Arafah et al. (44) and Furusawa et al. (46), who reported a similar high rate in patients with chronic lung diseases. The present study also found bronchial smooth muscle hypertrophy in 22/36 cats (61.1%) and hyperplasia or dilation of bronchial glands in 8/36 cats (22.2%). Mucosal edema, fibrosis, and vascular wall thickening were seen in 25/36 cats (69.4%), 25/36 (69.4%), and 11/36 (30.5%), respectively. This chronic inflammation may be linked to the ongoing effects of FCGS. These findings align with existing literature on respiratory diseases in cats, though in the majority of the existing studies, tissue samples were collected surgically or post-mortem rather than by tracheobronchoscopy (41, 47–49).

Study limitations include the small sample number of cats in the control group and limited sample size for histopathological analysis. Additionally, no histopathological examinations were performed on healthy cats due to ethical considerations and animal welfare concerns. To address this limitation in future research, the use of post-mortem samples from ethically sourced healthy controls could be considered, where appropriate, to better delineate disease-specific histopathological changes. Moreover, it should be noted that calicivirus screening was not performed in this cohort, representing a potential area for further investigation that could yield valuable information in future studies. A follow-up tracheobronchoscopy after treatment could be beneficial in assessing treatment effectiveness. While endoscopy is considered a safe diagnostic tool for lower airway conditions, it remains an invasive procedure for cats and is therefore not routinely recommended by the authors. However, in cases of persistent symptoms or multiple recurrences, a repeat tracheobronchoscopy may be required. Finally, future studies comparing the respiratory system in cats with respiratory disease and in cats with respiratory symptoms but without clinical signs of FCGS—both clinically via bronchoscopy and histopathologically—would be valuable in further clarifying potential associations.

## Conclusions

According to the results of the present study, cats with FCGS exhibit a high incidence of mild to severe chronic inflammatory lesions in the lower respiratory tract, albeit without obvious clinical signs. Therefore, in these cats, the lower respiratory system should be assessed to determine the extent and severity of inflammation, enabling more targeted and personalized treatment. While the systemic impact of FCGS has been recognized, larger studies are needed to clarify the mechanisms involved. Such researches will deepen the understanding of FCGS pathogenesis and support the development of more effective therapeutic approaches.

## Data Availability

The datasets presented in this study can be found in online repositories. The names of the repository/repositories and accession number(s) can be found in the article/supplementary material.
